# Prolonged Analgesia Following Short, Pulsed Meloxicam in Generalized Osteoarthritis: A Case Report and Focused Evidence Review

**DOI:** 10.7759/cureus.94026

**Published:** 2025-10-07

**Authors:** Akhtar Purvez, Mudhasir Bashir

**Affiliations:** 1 Clinical Research, Momentum Medical Research, Charlottesville, USA; 2 Clinical Sciences, Lincoln Memorial University DeBusk College of Osteopathic Medicine, Harrogate, USA; 3 Psychiatry and Behavioral Sciences, University of Virginia, Charlottesville, USA

**Keywords:** medication side effect, non-steroidal anti-inflammatory drugs (nsaids), nsaid complications, pulsed, renal

## Abstract

Oral nonsteroidal anti-inflammatory drugs (NSAIDs) are widely used to treat osteoarthritis (OA), but their risks rise with cumulative exposure across the gastrointestinal, cardiovascular, and renal systems. We report a 67-year-old individual with generalized OA, no other chronic illnesses, no medication allergies, and no history of trauma, who self-administers meloxicam 15 mg once daily for seven consecutive days each month. Each monthly pulse is followed by approximately three weeks of analgesia without NSAID use between pulses, and no adverse effects have been observed. Prior evidence suggests that NSAID analgesia peaks early while minor adverse events increase after longer continuous use; this patient’s real-world response supports a pragmatic, exposure-sparing approach. Although trials specifically testing fixed weekly or monthly NSAID pulses are lacking, careful patient selection and monitoring may allow short, structured courses that maintain function while minimizing cumulative risk.

## Introduction

Osteoarthritis (OA) is a chronic, degenerative disorder characterized by cartilage loss, osteophyte formation, and varying degrees of synovial and subchondral bone involvement, resulting in pain, stiffness, and functional limitation. Pharmacologic care often relies on nonsteroidal anti-inflammatory drugs (NSAIDs) to reduce pain and improve activity; however, the risk profile of NSAIDs spans the gastrointestinal (GI), cardiovascular (CV), and renal systems and scales with cumulative exposure (dose × duration) [1-3]. Contemporary guidance emphasizes using the lowest effective dose for the shortest possible duration and preferentially employing topical NSAIDs for knee OA when feasible, both to minimize systemic exposure and to individualize treatment according to risk [4,5].

The analgesic time course for oral NSAIDs in OA suggests that benefits emerge early and peak within the first two weeks, with diminishing marginal returns thereafter during uninterrupted use; concurrently, minor adverse events such as dyspepsia begin to rise after approximately four weeks of continuous therapy [6,7].

Within this framework, structured intermittent (“pulsed”) regimens - short, planned courses separated by NSAID-free intervals - represent a pragmatic attempt to capture early analgesic gains while curtailing cumulative risk. Although high-quality randomized data specifically evaluating fixed weekly or monthly oral NSAID pulses are lacking, the core principles guiding their cautious use derive from dose-duration toxicity relationships, broad evidence syntheses of NSAID effectiveness, and public health guidance that prioritizes risk minimization without sacrificing clinically meaningful function [8-12].

## Case presentation

A 67-year-old individual with generalized OA presented with chronic, widespread musculoskeletal pain involving the knees, hips, hands, and lumbar spine. There were no other chronic illnesses, no medication allergies, and no history of trauma or accidents. The patient explicitly reports no diabetes, hypertension, chronic kidney disease, peptic ulcer disease, or established CV disease. Concomitant medications are not used.

At presentation, the patient reported a pain of 8/10 using an 11-point Numeric Rating Scale, where 0 equals no pain and 10 equals the worst imaginable [13].

Analgesic self-management consisted of meloxicam 15 mg once daily for seven consecutive days each month (a "pulsed" regimen). Each monthly pulse provided approximately three weeks of pain relief, and no additional NSAID doses were needed or taken between pulses. Table 1 depicts pain level profiles after meloxicam ingestion.

**Table 1:** Progression of average self-recorded pain level score after meloxicam administration

Interval (weeks)	Pain score (0-10)
Week 1	3
Week 2	5
Week 3	8

The patient denied dyspepsia, melena, hematemesis, lower-extremity edema, dyspnea, chest pain, or elevation in home blood pressure readings since adopting this regimen.

Physical examination revealed normal vital signs (blood pressure 126/76 mmHg, heart rate 72 bpm, afebrile). Gait is mildly antalgic without assistive devices. There was no bony enlargement at the distal and proximal interphalangeal joints with Heberden and Bouchard nodes, mild hallux valgus, and crepitus at both knees. Range of motion is modestly reduced at the knees and hips without effusion or warmth. There is paraspinal tenderness over the lumbar facets without midline tenderness or radicular deficits. No synovitis is detected in the small joints of the hands or wrists. Neurologic screening is nonfocal.

Laboratory studies were obtained at baseline. Repeat studies, including electrolytes and renal function, performed at monthly intervals for three months, remained within reference ranges. Table 2 illustrates the laboratory values.

**Table 2:** Laboratory findings (with reference ranges) WBC: white blood cells; eGFR: estimated glomerular filtration rate; BUN: blood urea nitrogen; AST: aspartate aminotransferase; ALT: alanine transaminase

Test	Result	Reference range (typical adult)
Hemoglobin (g/dL)	13.8	12.0-16.0 (F); 13.5-17.5 (M)
Platelets (×10^9/L)	250	150-400
WBC (×10^9/L)	6.2	4.0-11.0
Serum creatinine (mg/dL)	0.9	0.7-1.3
eGFR (mL/min/1.73 m^2)	85	≥60
BUN (mg/dL)	15	7-20
Potassium (mmol/L)	4.3	3.5-5.1
Sodium (mmol/L)	140	135-145
AST (U/L)	19	10-40
ALT (U/L)	21	7-56
Urinalysis: protein	Negative	Negative
Urinalysis: blood	Negative	Negative

Plain radiographs demonstrated tricompartmental osteophytes and joint-space narrowing of both knees (Kellgren-Lawrence grade 2-3), mild joint-space narrowing of both hips with marginal osteophytes, interphalangeal OA of the hands with osteophyte formation and subchondral sclerosis, and multilevel lumbar facet arthropathy without spondylolisthesis. No erosions are seen. These findings are consistent with generalized OA.

Assessment

Generalized OA with satisfactory symptom control achieved using a short, repeated meloxicam pulse. The safety profile to date is acceptable in a low-risk individual with reassuring blood pressure, renal function, and GI status.

## Discussion

**Figure 1:**
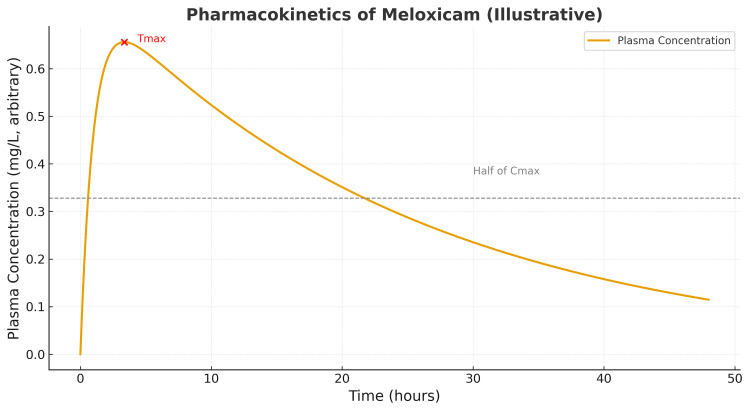
Meloxicam pharmacokinetic curve in health adults

With a known pharmacokinetic pattern of meloxicam in healthy adults, one would assume that the relief would begin quickly after ingestion, peak at one to two hours, and then drop over the course of the next 40-50 hours (Figure 1).

However, meta-analyses in knee OA demonstrate that oral NSAID analgesic effects tend to peak within the first two weeks of therapy and subsequently wane. In contrast, minor GI adverse events begin to rise after approximately four weeks of continuous dosing. Randomized comparisons of continuous versus intermittent (flare-based) COX‑2 selective NSAID use have shown broadly similar outcomes, with a modest trend favoring continuous dosing for flare prevention; both regimens were generally well tolerated. No randomized trials were found that specifically evaluated a fixed monthly seven-day meloxicam pulse [6,7,9,12].

Given that most NSAID-related adverse events, including GI, CV, and renal complications, are dose- and duration-dependent, strategies that reduce cumulative exposure are expected to improve safety, provided analgesia remains adequate. In practice, this translates to carefully monitored intermittent dosing in appropriately selected patients, alongside nonpharmacologic care and topical NSAIDs where feasible [1-3].

Practical and monitored approach

A practical and monitored approach begins with shared decision-making that transparently balances the expected analgesic benefits against exposure-dependent toxicities. The patient should be informed that steady daily NSAID use confers cumulative risk across GI, CV, and renal domains, and that a short, structured monthly pulse is being considered to reduce total drug exposure while preserving function. This discussion should explicitly acknowledge the hypothesis‑generating nature of a seven‑day monthly meloxicam course and the absence of randomized trials validating this exact regimen, while emphasizing that the rationale is grounded in the established dose‑ and duration‑response relationship for NSAID harms [1-3].

Patient selection is essential. Candidates are those with OA who have inadequate relief from nonpharmacologic care and topical NSAIDs, lack major cardiometabolic or renal comorbidity, and can adhere to monitoring. For such patients, a pulsed regimen of meloxicam 15 mg once daily for five to seven days during flares or as a planned once‑monthly course can be framed as an exposure‑sparing strategy that remains consistent with guideline principles of using the lowest effective dose for the shortest possible duration. Counseling should also reinforce joint-protective exercises, weight management, and topical therapies as foundational measures, reserving oral medications for breakthrough symptoms or defined cycles of activity-related worsening [4,5].

Clinicians should set expectations using the observed time course of NSAID effects in OA: analgesia typically peaks within two weeks and attenuates with continued dosing, whereas minor adverse events begin to rise after roughly four weeks of uninterrupted therapy. In this context, short pulses that capture the early benefit window without extending into the period when adverse events accumulate are a plausible way to balance efficacy and safety. The monthly‑pulse approach should therefore be paired with scheduled reassessment of pain intensity, function, and rescue‑medication use, with permission to shorten or skip pulses during lower‑symptom months and to intensify nonpharmacologic modalities during higher‑symptom periods [6-8].

Because most rigorous evidence evaluates daily or near‑daily dosing, clinicians should document that effect sizes for oral NSAIDs are generally small to moderate and that some chronic pain populations (for example, chronic low back pain) experience only modest, sometimes clinically marginal, improvements. Network meta-analyses and contemporary reviews underscore heterogeneity of response and reinforce the principle that more prolonged exposure does not guarantee sustained benefit. Within this evidentiary landscape, a pulsed plan is reasonable when symptom relief is demonstrably clustered in the early treatment window and when the patient values a lower pill burden and risk profile, provided that careful follow-up confirms the stability of renal function, blood pressure, and GI tolerance [9-12].

Risk mitigation should be individualized and proactive. For GI protection, consider a proton pump inhibitor in patients aged 65 or older, those with prior ulcer disease, or those receiving concomitant anticoagulants, antiplatelets, corticosteroids, or serotonergic agents. Where clinically appropriate, evaluate and eradicate *Helicobacter pylori* to reduce ulcer risk. Avoid multiple NSAIDs and unnecessary aspirin use, and educate patients about alarm symptoms such as melena, hematemesis, progressive dyspepsia, new edema, dyspnea, or chest pain that warrant immediate evaluation. These strategies, derived from reviews of arthritis pharmacotherapy, dyspepsia literature, landmark analyses of NSAID GI toxicity, and expert consensus statements, can substantially curtail preventable harm when paired with lower cumulative exposure [14-17].

Monitoring should combine laboratory testing and clinical surveillance timed to the pharmacologic exposure. Obtain baseline and early follow‑up serum creatinine with estimated glomerular filtration rate and electrolytes, especially potassium, after the first pulse, with periodic blood pressure checks thereafter. Guard against the “triple whammy” of angiotensin-converting-enzyme (ACE) inhibitor or angiotensin‑receptor blocker plus diuretic plus NSAID; maintain adequate hydration; and reassess every three to six months to confirm that pulses remain effective and well tolerated. Observational data on work‑age populations illustrate how GI events and costs escalate with sustained exposure, contemporary peptic‑ulcer reviews detail the mechanisms and timelines of harm, and FDA labeling highlights that CV risk can appear early and rise with duration - points that support a structured, exposure‑sparing regimen with clear stopping rules and a bias toward the shortest necessary course [18,19].

Patient perspective

The patient values three weeks of relief following a short, seven-day course each month and prefers this approach to daily medication owing to the lower pill burden and perceived safety.

## Conclusions

Short, structured “pulsed” meloxicam of 15 mg daily for seven days monthly provided this low-risk patient with about three weeks of analgesia per pulse and no observed GI, renal, CV, or hemodynamic complications during monitoring. This pattern fits the known early peak and later attenuation of NSAID benefit while limiting cumulative exposure that drives toxicity.

Within shared decision-making, such exposure-sparing pulses can align with guidance to use the lowest effective dose for the shortest duration, alongside nonpharmacologic care and topical NSAIDs when feasible. Although fixed weekly or monthly NSAID pulses lack randomized trial validation, cautious, monitored implementation in carefully selected patients is reasonable, with clear stopping rules and periodic checks of renal function, blood pressure, and GI tolerance.
